# Transcriptomics and differential gene expression in *Whitmania pigra* (Annelida: Clitellata: Hirudinida: Hirudinidae): Contrasting feeding and fasting modes

**DOI:** 10.1002/ece3.5074

**Published:** 2019-03-18

**Authors:** Muhammad Salabat Khan, De‐Long Guan, Sebastian Kvist, Li‐Bin Ma, Juan‐Ying Xie, Sheng‐Quan Xu

**Affiliations:** ^1^ College of Life Sciences Shaanxi Normal University Xi'an China; ^2^ Department of Ecology and Evolutionary Biology University of Toronto Toronto Ontario Canada; ^3^ Department of Natural History Royal Ontario Museum Toronto Ontario Canada; ^4^ School of Computer Science Shaanxi Normal University Xi'an China

**Keywords:** anticoagulants, differential gene expression, Hirudinea, Hirudinidae, metabolic pathways, transcriptomics, *Whitmania pigra*

## Abstract

The medicinal utility of leeches has been demonstrated through decades of use in modern hospital settings, mainly as relievers of venous congestion following flap or digit replantation surgery. In the present study, we sequence and annotate (through BLAST‐ and Gene Ontology‐based approaches) the salivary transcriptome of the nonblood feeding hirudinid *Whitmania pigra* and assess the differential gene expression of anticoagulation factors (through both quantitative real‐time PCR [qRT‐PCR] and in silico‐based methods) during feeding and fasting conditions. This was done in order to evince the diversity of putative anticoagulation factors, as well as estimate the levels of upregulation of genes immediately after feeding. In total, we found sequences with demonstrated orthology (via both phylogenetic analyses and BLAST‐based approaches) to seven different proteins that have previously been linked to anticoagulatory capabilities—eglin C, bdellin, granulin, guamerin, hyaluronidase, destabilase I, and lipocalin. All of these were recovered from leeches both in the fasting and in the feeding conditions, but all show signs of upregulation in the feeding leeches. Interestingly, our RNA‐seq effort, coupled with a hypergeometric test, indicated that the differentially expressed genes were disproportionately involved in three main immunological pathways (endocytosis, peroxisome regulation, and lysosome regulation). The results and implications of the finding of anticoagulants in this nonblood feeding leech and the putative upregulation of anticoagulation factors after feeding are briefly discussed in an evolutionary context.

## 1 INTRODUCTION

Anticoagulation factors work directly, or in concert, to antagonize coagulation cascades that normally act to induce thrombus formation following trauma to the blood vessels. As a result of the anticoagulation capabilities, these factors are also commonly employed in hospital settings—for example, to prevent blood clot formation following stroke (Sanama & Guinet, 2011; Sandercock, Counsell, & Kane, 2015; Weitz & Bates, 2005)—and they are currently available in pill form or as intravenous medication (Garcia, Libby, & Crowther, 2010). These anticoagulants also occur naturally in blood feeding organisms, making the animals themselves of utility to modern medicine. For example, for millennia, leeches have been leveraged for their blood feeding nature and possession of potent anticoagulants (Siddall, Min, Fontanella, Phillips, & Watson, 2011 and references therein) and their frequency of use in medicine continues to increase (Mory, Mindell, & Bloom, 2000). Although hirudin, which binds bivalently to both the exosite and the catalytic site of thrombin thereby preventing the transformation of fibrinogen into fibrin, is arguably the most well‐known leech anticoagulation factor, the salivary peptide repertoires of leeches may include over 20 compounds known to be involved in preventing coagulation of blood (Kvist, Brugler, Goh, Giribet, & Siddall, 2014). These include factor Xa inhibitors, thrombin inhibitors, inhibitors of platelet aggregation, protease inhibitors, and leukocyte elastase inhibitors, to name a few. In addition, some of these anticoagulation factors are extremely potent, active in picomolar concentrations (Dang & Cera, 1994), and some bind irreversibly to their target proteins, suggesting the selective advantage that they confer to the leech, to enable extended periods of feeding.

With an increase in evidence from independent investigations, we now believe that the ancestral leech was blood feeding, or at least possessed anticoagulation factors that presumably aided in the leeches' feeding biology (Siddall, Brugler, & Kvist, 2016; Siddall et al., 2011). If the ancestral leech was blood feeding, one could reasonably expect to find signatures of this ancestry in the transcriptomic makeup of nonblood feeding leeches, as well as the finding of orthologous anticoagulants in both leeches that share an ancient common ancestor and those that share more derived common ancestors. Indeed, both of these scenarios have been shown to be true for leeches, with the possession of leech antiplatelet proteins (LAPP) by the nonblood feeding leech, *Helobdella robusta* Shankland, Bissen & Weisblat, 1992 (Kvist, Sarkar, & Siddall, 2011), and the possession of hirudin orthologues in glossiphoniid and hirudinid leeches, the common ancestor of which existed relatively early in leech evolution (Siddall et al., 2016). However, nonblood feeding leeches from across the phylogeny need to be queried in order to robustly infer if these possess anticoagulation factors. This will both shed light on the diversity and evolution of leech anticoagulation factors more generally and will help elucidate the specific feeding habit of the ancestral leech. In addition, our knowledge regarding the expression of anticoagulants during periods of digestion (i.e., fasting) and their potential upregulation during feeding is still almost nonexistent.

The Asian freshwater leech *Whitmania pigra *(Whitman, 1884) is nonblood feeding, despite the placement of this genus within the family Hirudinidae (Phillips & Siddall, 2009), which also includes the European medicinal leech, *Hirudo medicinalis *(Linnaeus, 1758) and several other blood feeding species. Instead, the leech is considered macrophagous, suggesting that it commonly swallows or takes bites out of prey sources (Sawyer, 1986). In traditional Chinese medicine, *W. pigra *is known to promote blood circulation and relieve blood clotting (Jia‐Xin, Xing‐Chang, & Qiu‐Hai, 1994; Zhong, Yang, & Cui, 2008) and it can therefore be reasonably deduced that this leech may possess anticoagulation factors despite of its nonblood feeding nature. However, large‐scale transcriptomic evidence for such a claim is sorely lacking, despite the previous sequencing of a transcriptome from an individual of *W. pigra *(Liu et al., 2018); that study focused on environmental adaptations of leeches, rather than the anticoagulant repertoire. To clarify whether or not this nonblood feeding leech secretes anticoagulants, which anticoagulants, and to shed light on the timing of their putative expression, the present study sequenced full body transcriptomes of *W. pigra*, both during feeding and during periods of digestion (fasting), followed by differential gene expression analyses.

## 2 MATERIAL AND METHODS

### 2.1 Sample preparation

**Figure 1:**
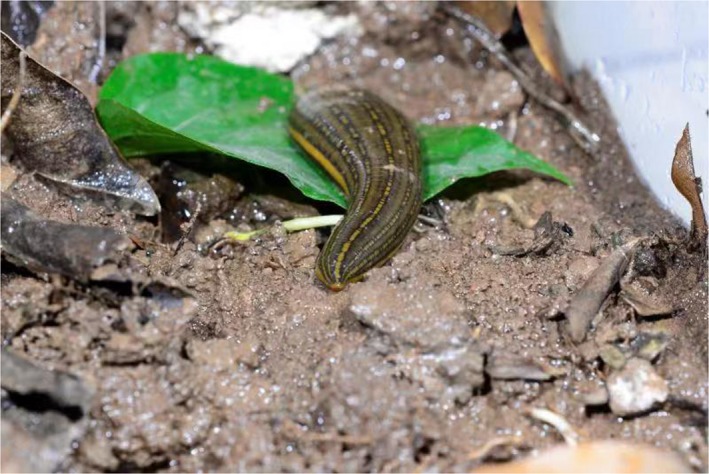
Live habitus of *Whitmania pigra*. Photograph by M. Salabat Khan

Specimens of *W. pigra* (see Figure 1) were provided by the Institute of Zoology Lab, Shaanxi Normal University, China, with live snails delivered regularly as a food source. The specimens were divided into two test groups: fasting (*n* = 3) and feeding (*n* = 3). After allowing the leeches to acclimatize to their new environment for 1 month, specimens in the fasting group were deprived of food for 2 weeks, whereas specimens in the feeding group were continuously fed live snails. Prior to RNA extraction, all leeches were rinsed sequentially in a bleach solution and deionized water to reduce the risk of contamination by surface bacteria. The samples were stored in a liquid nitrogen facility until RNA extraction.

### 2.2 RNA isolation and library preparation

Total RNA was extracted both from the whole body of each of the specimens and also directly from salivary tissues using the Qiagen RNA Kit (Qiagen Biotechnology, Hilden, Germany) following the manufacturer's instructions. RNA degradation and/or putative contaminations were checked by gel electrophoresis on a 1% agarose gel. Further, the purity, concentration, and integrity of the RNA samples were tested using a RNA Nano 6000 Assay Kit on an Agilent Bioanalyzer 2100 system (Agilent Technologies, CA, USA). Libraries were constructed for samples that passed initial degradation tests. In total, 3 μg of total RNA for each sample was used for the RNA sample preparations. Briefly, eukaryotic mRNA was enriched using poly‐T oligo‐attached magnetic beads and subsequently randomly fragmented in fragmentation buffer (Biomarker, Beijing, China). Then, mRNA was separated from the total RNA sample by divalent cations under high temperature and the mRNA was used as the template for cDNA synthesis using random hexamers. Second‐strand cDNA fusion was achieved using DNA polymerase I with the addition of RNase, and double‐stranded cDNA was thereafter purified using AMPure XP beads (Beckman Coulter, California, USA). Complementary DNA fragments of preferentially 150–200 bp length were selected with the AMPure XP system. Index‐coded sample clustering was achieved on a cBot Cluster Generation System using TruSeq PE Cluster Kit v3‐cBot‐HS (Illumina Inc., California, USA). After cluster formation, paired‐end sequencing was performed on the Illumina HiSeq 4000 platform.

### 2.3 Quality control

The raw data were principally treated with in‐house perl scripts. At this stage, adapter sequences were removed and reads including poly‐N regions, as well as low‐quality reads (PHRED scores below 30), were eliminated from the dataset. Simultaneously, GC content of the clean data was calculated. All downstream analyses were based on the resulting, quality‐controlled data.

### 2.4 Transcriptome assembly and annotation

A reference genome‐guided assembly against the genome of *H. robusta *was performed for each of the paired‐end transcriptomes using Trinity (Grabherr et al., 2011), and applying default settings. Transdecoder ver. 5 (https://github.com/TransDecoder/TransDecoder) was used to identify putative coding regions within transcripts. Initial transcript identities (some of which were later confirmed by phylogenetic analyses) were assessed by BLASTing (using the BLASTn algorithm) against the following databases: GenBank nr (nonredundant sequence database), Pfam, and Swiss‐Prot. Gene ontology terms were then established using the *topGO* R package (Aibar, Fontanillo, Droste, Rivas, & J., 2015), with analyses based on the Kolmogorov–Smirnov test (Antoneli, Passos, Lopes, & Briones, 2018).

### 2.5 Identification of differentially expressed genes

Following the results from the RNA‐seq data, in silico differential expression analysis of the two conditions (fasting and feeding) was performed using DESeq ver. 1.10.1 (Anders & Huber, 2010) in R (R Core Team, 2017) for the six transcriptomes resulting from the RNA sequencing. The Pearson correlation coefficient was used as an indicator of biological repeat correlation between the different samples. The screening criteria involved fold changes ≥2 or ≤0.5, and false discovery rate (FDR) <10% (see Anders & Huber, 2010), using normalized counts of transcripts as input. The resulting *p*‐values were adjusted using the Benjamini and Hochberg (Benjamini & Hochberg, 1995) procedure for controlling for false discovery rates. Genes for which an adjusted *p*‐value ≤ 0.05 was recovered were interpreted as differentially expressed.

As corroboration of the DESeq analysis, we performed SYBER GREEN‐based, quantitative real‐time PCRs (qRT‐PCR) of select proteins, in order to robustly infer the differential gene expression that was proposed by the in silico method. Reverse transcription and cDNA synthesis used 300 ng of total mRNA. Primers (Supporting Information Table S1) were designed from the whole‐body transcriptome data employing Primer‐BLAST (Ye et al., 2012). Seven putative anticoagulant proteins were chosen for qRT‐PCR validation (eglin C, bdellin, granulin, guamerin, hyaluronidase, destabilase I, and lipocalin). The qRT‐PCR was run on a CFX96 real‐time PCR detection system (Bio‐Rad, California, USA), and statistical analysis of the results was performed using a two‐way ANOVA. A total of six biological replicates (three for fasting leeches and three for recently fed leeches) were used to corroborate the results. Expression levels of target genes (the seven putative anticoagulation factors) were calculated with respect to mean RT‐PCR, by quantifying the cycle threshold (*C*_T_) value. The Δ*C*_T_ method performs pairwise, relative expression level comparisons of genes, and this method was used herein for normalization of qRT‐PCR data.

A pathway annotation analysis of the differentially expressed genes was employed via a BLAST‐based search against the Kyoto Encyclopaedia of Genes and Genomes (KEGG), and a hypergeometric test (Fisher's exact test; see, e.g., Goeman & Bühlmann, 2007) was used to identify any potential skew of the differentially expressed genes toward certain biological pathways (e.g., immunology).

### 2.6 Amino acid alignments and phylogenetic analyses

Translated nucleotide sequences for each of the recovered anticoagulation factors were aligned together with a single “archetypal” anticoagulant. The online version of MAFFT ver. 7 (Katoh & Standley, 2013) was employed with default settings, and the results were visualized using Jalview ver. 2.10.2 (Waterhouse, Procter, Martin, Clamp, & Barton, 2009). From this alignment, percent similarity was calculated and putative conservation of the cysteines, involved in disulfide bonds and protein folding, was estimated. The online platform for SignalP ver. 4.1 (Petersen, Brunak, Heijne, & Nielsen, 2011) was used to estimate the presence of a signal peptide for each of the putative anticoagulant sequences.

In addition, phylogenetic analyses were performed for a larger subset of sequences (these were recovered from previous leech anticoagulant studies (e.g., Min, Sarkar, & Siddall, 2010; Kvist, Min, & Siddall, 2013; Kvist et al., 2014; Siddall et al., 2016; Tessler, Marancik et al., 2018) including several putative orthologs beyond the archetypal sequences (the putative guamerin sequence from *W. pigra *was aligned with several known, putatively paralogous factor Xa inhibitors derived from leeches, in order to tease out the most likely ortholog for this sequence). Phylogenetic analyses were performed in order to robustly infer orthology between the newly derived sequences and the archetypal anticoagulants. Again, MAFFT ver. 7 was employed with default settings. To maximize homology and avoid detrimental effects by the partial nature of the comparative transcriptomic data, the alignments were cut to reflect the coverage of the specific genes for the *W. pigra *data. Phylogenetic analyses were performed in TNT ver. 1.5 (Goloboff, Farris, & Nixon, 2008) under the maximum parsimony criterion with gaps treated as missing data. For this purpose, 1,000 new technology replications with five rounds of ratcheting were employed, stipulating that the best tree should be found 10 times before terminating the search (“hits 10”). The results where then subjected to TBR branch swapping using the “bbreak” command. Support values were estimated using 100 rounds of standard bootstrapping. All trees were left unrooted due to the absence of an obvious outgroup for leech anticoagulants.

## 3 RESULTS

In total, we recovered 41.35 Gb of clean data from all specimens combined and predicted a total of 22,632 genes with mean length of 3,397 bp and with 95.14% of transcripts showing PHRED scores above 30. The mean GC content for the combined pool of fasting and feeding individuals was 43.17%. Sample details are provided in Table 1, and detailed description of transcript lengths is shown in Supporting Information Figure S1.

**Table 1:** Sample details resulting from the RNA‐seq approach. WP‐1, WP‐2, and WP‐3 represent leeches in fasting mode, whereas WP‐4, WP‐5, and WP‐6 represent leeches in feeding mode

Sample ID	Raw reads	Clean reads	Raw bases (Gb)	Clean bases (Gb)	Effective rate (%)	Q20 (%)	Q30 (%)	GC (%)
WP‐1	46835484	44106896	7.03	6.62	94.17	98.17	95.36	43.71
WP‐2	48362626	46625030	7.25	6.99	96.41	97.96	94.95	42.90
WP‐3	51222768	49069134	7.68	7.36	95.80	98.01	95.07	42.73
WP‐4	47602144	45290524	7.14	6.79	95.14	98.18	95.42	43.43
WP‐5	46859934	44655484	7.03	6.70	95.30	98.12	95.33	42.91
WP‐6	47742030	45942546	7.16	6.89	96.23	97.85	94.73	43.37

Note

The Q20 and Q30 columns indicate the percentage of overall base calls with a PHRED score ≥20 or ≥30, respectively; the GC column indicates the GC content across all reads.

### 3.1 GO analysis

**Figure 2:**
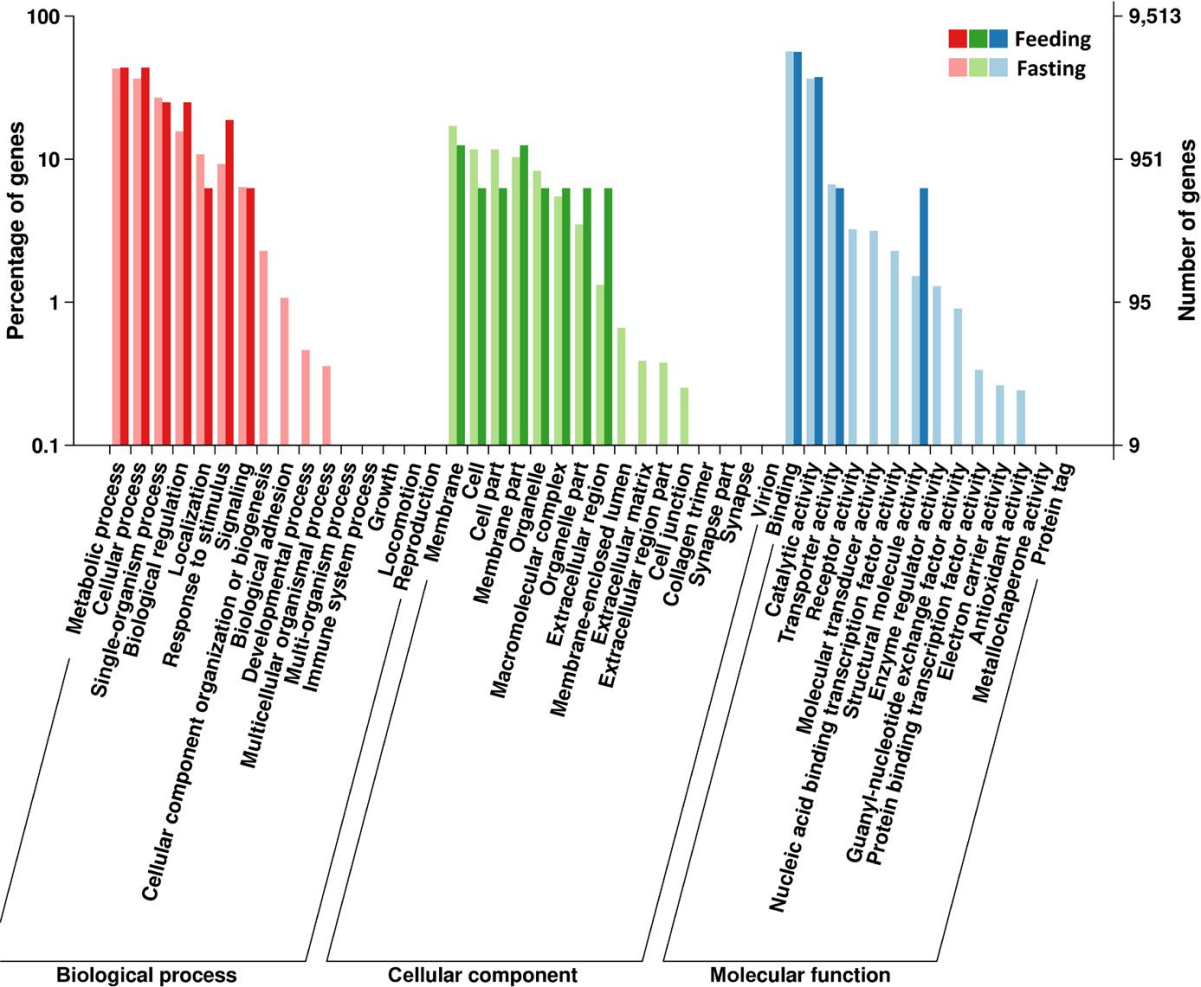
Results from the gene ontology analysis (examined using topGO), illustrating the log scale proportional devotion of the salivary transcripts to various processes regarding biological process, cellular component, and molecular function

Functional categories (cellular component, molecular function, and biological process) for gene ontology statements were assigned to each of the 22,632 gene regions (see Figure 2). In the biological process category, the four most common processes were metabolic process, cellular process, single‐organism process, and biological regulation. Within the cellular component category, the most enriched components were membrane, cell, cell part, organelle, and membrane part. In the molecular function category, the most highly expressed functions were binding activity, catalytic activity, transporter activity, and receptor activity.

### 3.2 Anticoagulant alignments

Despite the nonblood feeding nature of *W. pigra*, we found transcripts that show significant BLAST hits (e‐values below E^−5^) against seven different proteins that have previously been linked to anticoagulation. These were eglin C, bdellin, granulin, guamerin, hyaluronidase, destabilase I, and lipocalin (Table 2). Each of these transcripts was reciprocally BLASTed against GenBank nr to ensure that they did not match any other protein at a lower e‐value.

**Table 2:** Summary statistics for the seven putative anticoagulation factors found in the transcriptome of *Whitmania pigra*, as well as the details of the differential expression between feeding and fasting modes

Name	Biological actions	Normalized count (Fasting)	Normalized count (Feeding)	Log2 fold change	Up/Down
Lipocalin	Platelet aggregation inhibitor	2.357	749.58	2.64447	UP
Granulin	Thrombin inhibitor, Elastase inhibitor	35.365	171.441	1.71898	UP
Hyaluronidase	Hyaluronic acid cleavage	10.548	29.450	1.2631	UP
Bdellin	Trypsin and plasmin inhibitor	46.207	83.891	0.30256	UP
Eglin	Inhibitor of elastase/chymotrypsin	2.866	37.452	3.09321	UP
Destabilase	Fibrin depolymerase factor	5,062.76	18,880.8	1.34167	UP
Guamerin	Elastase inhibitor	175.43	1,145.28	1.2631	UP

Note

DESeq was used to calculate the normalized counts for transcripts, and these counts were used to estimate the Log2 fold change.

**Figure 3:**
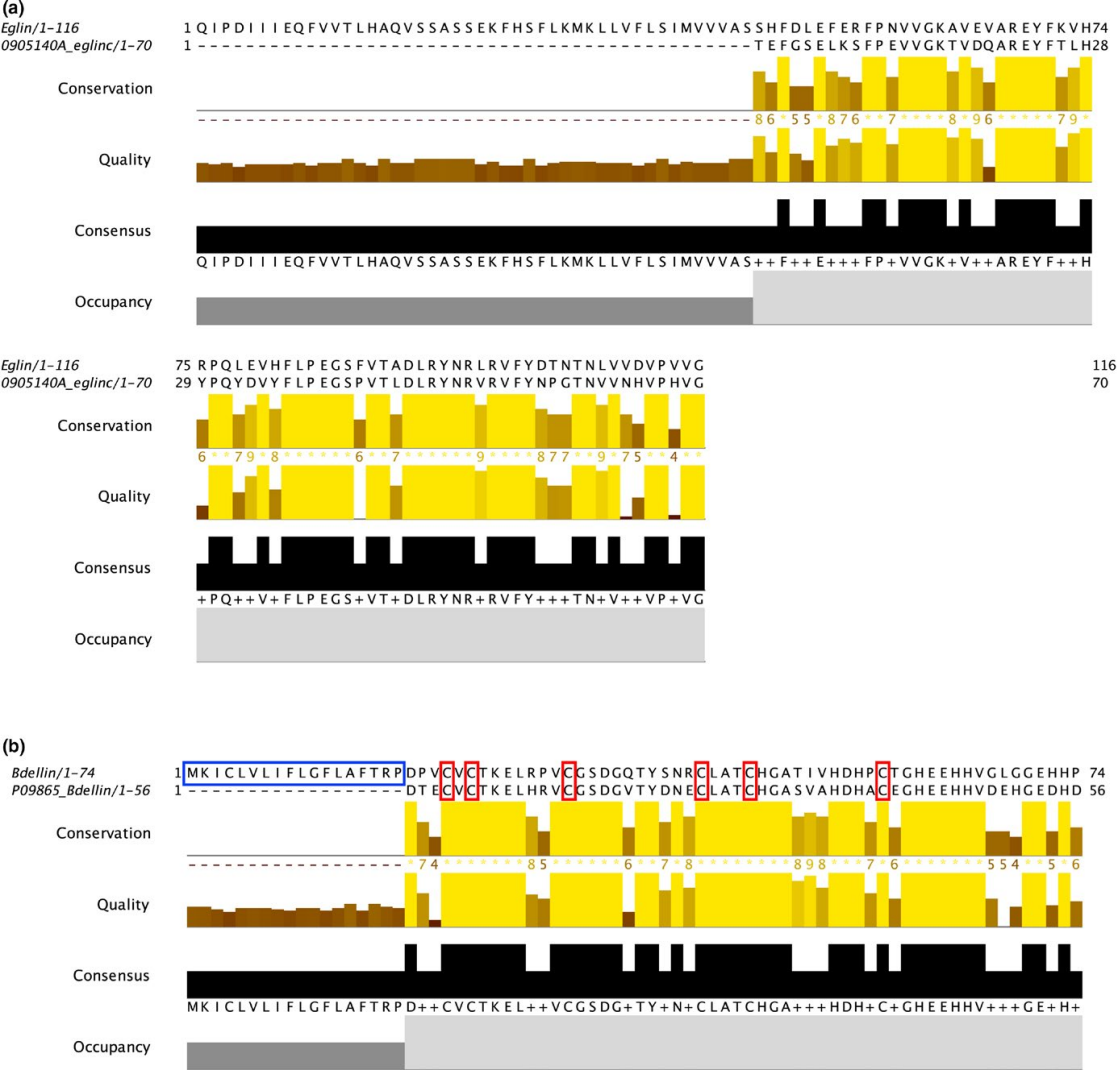
MAFFT‐based alignment of the amino acid sequences for (a) eglin C and (b) bdellin putative orthologues. The newly acquired, *Whitmania pigra*‐derived sequence is shown above, and the “archetypal” sequence below in the alignments. Blue boxes denote the predicted signal peptide region, and red boxes denote fully conserved cysteine residues

For eglin C, the amino acid alignment (Figure 3a) of the newly acquired sequence with the archetypal variant (GenBank accession number 0905140A; Knecht et al., 1983), originally isolated from an unidentified leech, showed 61% similarity between shared amino acid positions. Both sequences are devoid of cysteine residues, suggesting the nonfolding nature of the protein.

The amino acid alignment for bdellin (Figure 3b) indicated that the sequence derived from *W. pigra *shares 70% similarity (in positions without gaps) with the archetypal anticoagulant, originally isolated from salivary tissue of *H. medicinalis*; GenBank accession number P09865 (Fink et al., 1986). Of particular import, the six cysteine residues of the mature peptide (i.e., downstream from the signal peptide) that are responsible for the disulfide bonds and, therefore, the folding capability of the protein are fully conserved between the two sequences.

**Figure 4:**
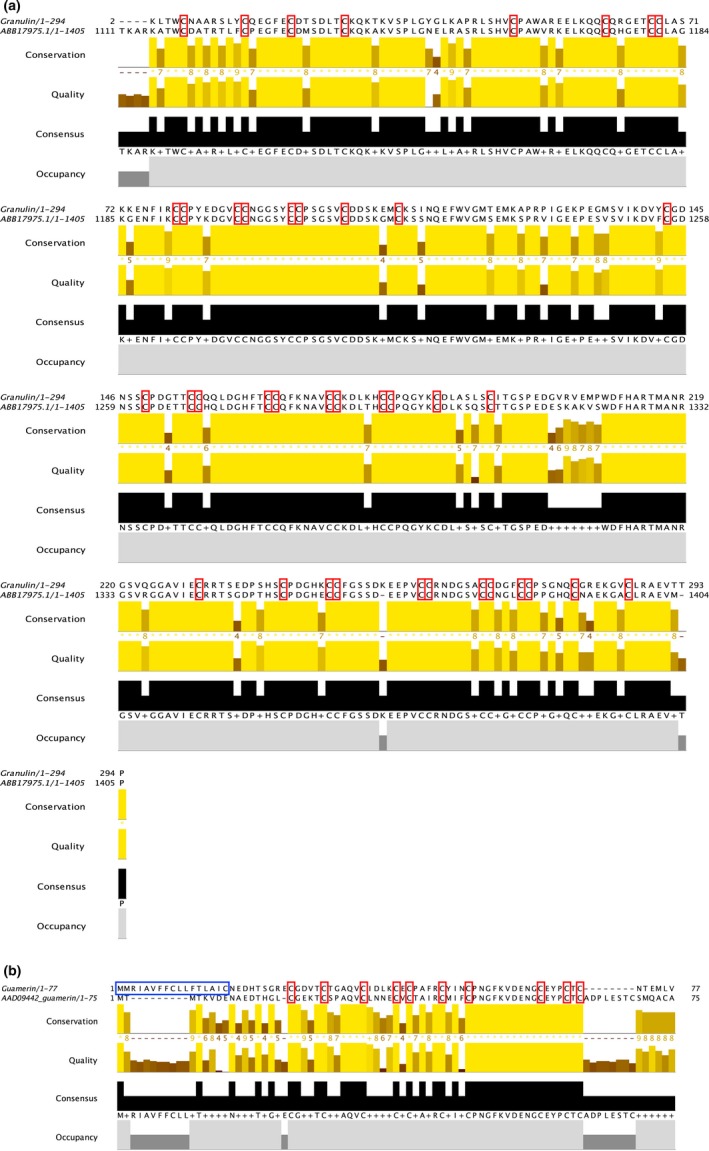
MAFFT‐based alignment of the amino acid sequences for (a) granulin and (b) guamerin putative orthologues. The newly acquired, *Whitmania pigra*‐derived sequence is shown above, and the “archetypal” sequence below in the alignments. Blue boxes denote the predicted signal peptide region, and red boxes denote fully conserved cysteine residues

The amino acid alignment for our granulin sequence (Figure 4a) together with the archetypal anticoagulant (GenBank accession number ABB17975; see He & Bateman, 2003) suggested that only a partial sequence had been generated for the present study; we recovered only 294 out of the estimated 1,405 amino acids. Regardless, for shared amino acid sites, our sequence shows 80% sequence similarity with progranulin derived from *H. medicinalis*. Within the homologous region of the mature peptide, the 40 cysteine residues (including 12 sets of two consecutive cysteines) are fully conserved between the sequences.

For guamerin, the amino acid alignment (Figure 4b) shows 43% amino acid identity between the sequence from *W. pigra *and the archetypal sequence, derived from *Hirudo nipponia *(GenBank accession number AAD09442; Jung, Kim, Ha, Joe, & Kang, 1995). Despite of this relatively low similarity, 10 out of the 12 cysteine residues show conservation and a relatively long stretch of homopolymers exist in the alignment (5′‐CPNGFKVDENGCEYPCTC‐3′).

**Figure 5:**
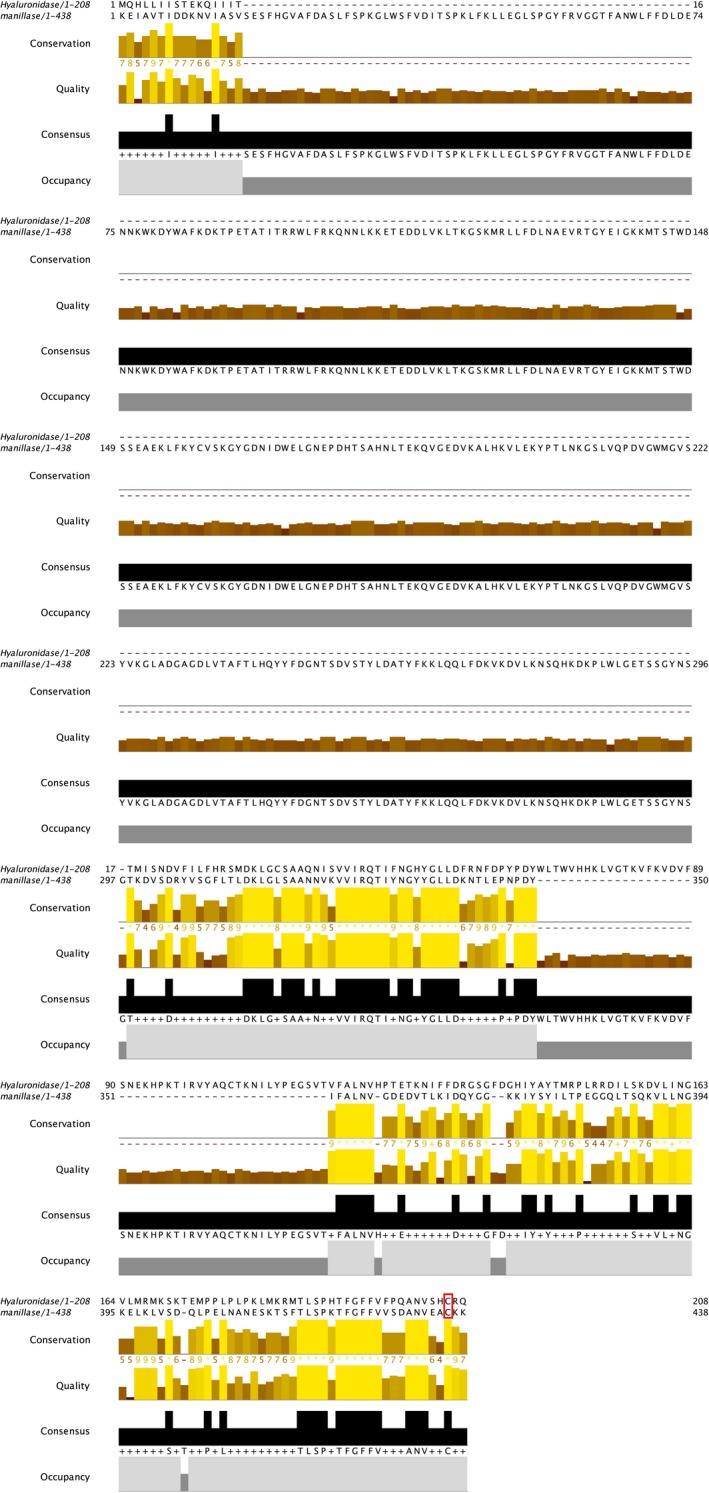
MAFFT‐based alignment of the amino acid sequences for the hyaluronidase manillase. The newly acquired, *Whitmania pigra*‐derived sequence is shown above, and the “archetypal” sequence below in the alignment. The red boxes denote the only fully conserved cysteine residue. Note the long gapped region toward the 5′‐end of the sequence (see text for further details)

For hyaluronidase (Figure 5), the archetypal anticoagulant sequence for manillase, recovered from the US Patent application US7, 049, 124 B1, was aligned with the newly generated sequence. Manillase has demonstrated endoglucuronidase/hyaluronidase activity and is one of the few leech‐derived proteins of this family of genes. Oddly, the alignment includes two large regions of gaps, suggesting either that the assembly may have inadvertently created chimeric sequences or that two insertion events have happened for these sequence (one in the archetypal sequence and one in the newly generated sequence). Regardless, for shared amino acid positions, the two sequences show 40% similarity. Of the four cysteine residues present in the archetypal sequence, only a single residue is fully conserved (note that two cysteines occur in the region of gaps). Previous studies have found similar values of amino acid similarity within this relatively long molecule (Kvist et al., 2014).

**Figure 6:**
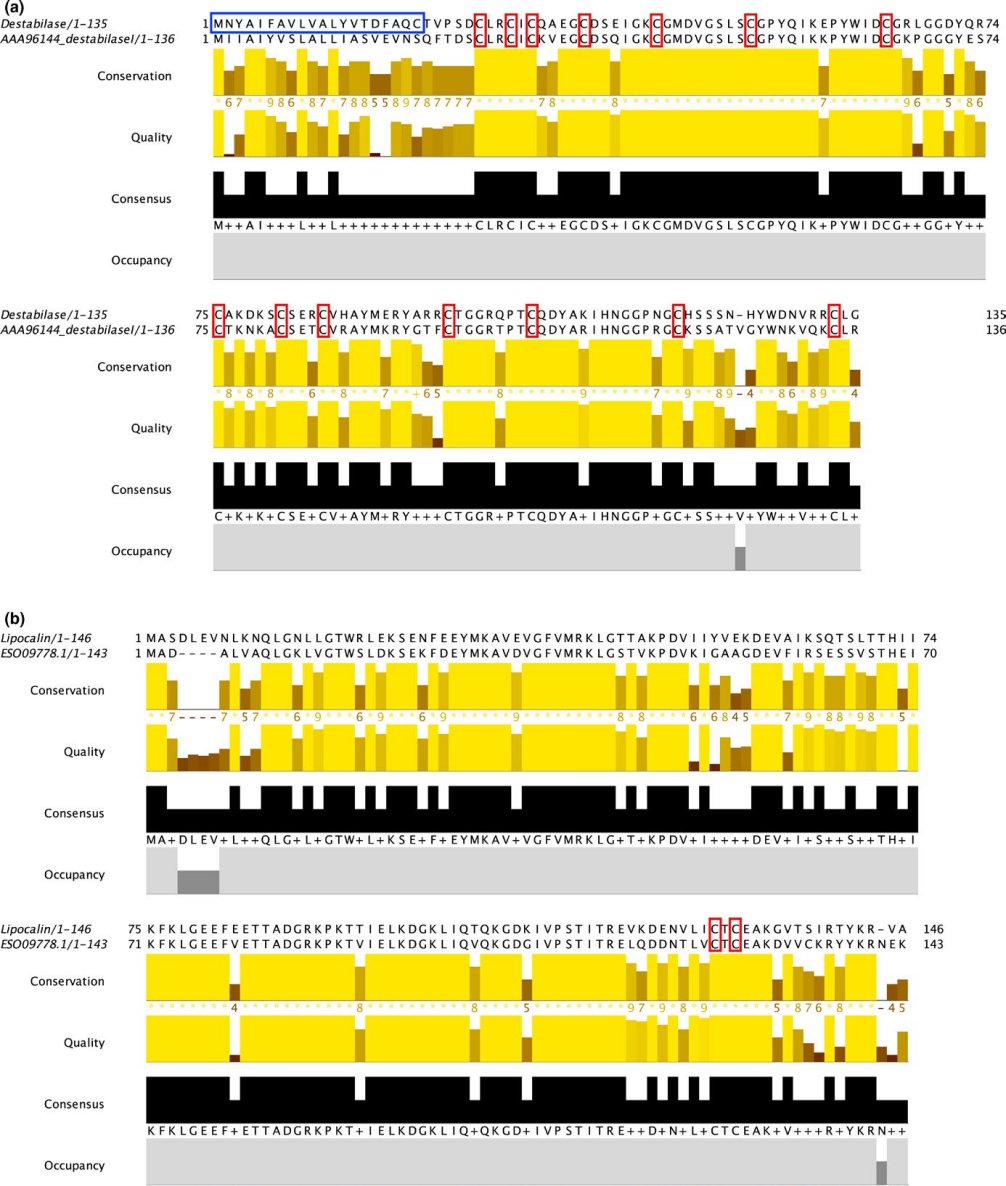
MAFFT‐based alignment of the amino acid sequences for (a) destabilase I and (b) lipocalin putative orthologues. The newly acquired, *Whitmania pigra*‐derived sequence is shown above, and the “archetypal” sequence below in the alignments. Blue boxes denote the predicted signal peptide region, and red boxes denote fully conserved cysteine residues

Our newly generated sequence of putative destabilase I shows 62% amino acid identity with the archetypal sequence (GenBank accession number AAA96144; Zavalova et al., 1996) isolated from *H. medicinalis *(Figure 6a). The 14 cysteine residues that are present in both sequences show full conservation.

Finally, in the amino acid alignment for lipocalin (Figure 6b), including our newly generated sequence and an archetypal sequence isolated from *H. robusta *(GenBank accession number ESO09778; Simakov et al., 2013), the two sequences show 68% amino acid identity. In addition, two out of the three cysteine residues present in the archetypal sequence (note the uneven number of residues) are conserved in the alignment and relatively long stretches of homopolymers are present, suggesting orthology between the sequences.

### 3.3 Phylogenetic trees

**Figure 7:**
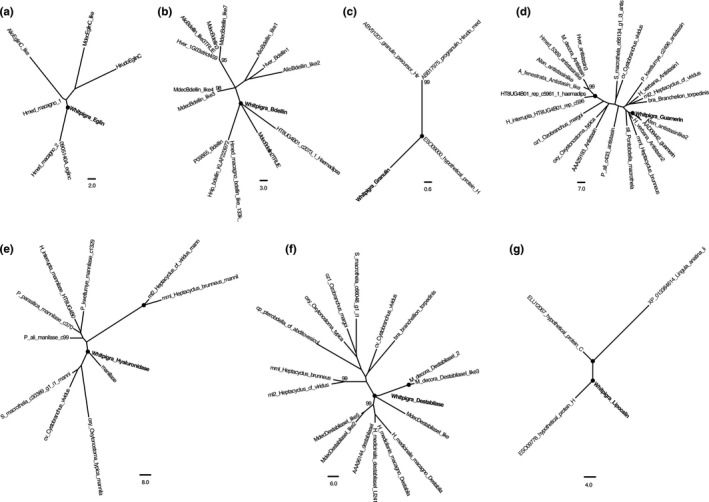
Single most parsimonious trees for leech‐derived putative orthologues of (a) eglin C (length: 171, CI: 0.971, RI: 0.792), (b) bdellin (length: 220, CI: 0.879, RI: 0.797), (c) granulin (length: 173, CI: 1.00, RI: 1.00), (d) antistasin (length: 1,093, CI: 0.837, RI: 0.649), (e) manillase (length: 906, CI: 0.959, RI: 0.825), (f) destabilase (length: 521, CI: 0.769, RI: 0.618), and (g) lipocalin (length: 199, CI: 1.00, RI: 1.00). The newly acquired sequences are denoted in bold font. CI: consistency index; RI: retention index.

The phylogenetic trees resulting from the analyses of the newly sequenced putative anticoagulant sequences and their potential allies are presented in Figure 7a–g.

For the eglin C tree (Figure 7a; length: 171, consistency index [CI]: 0.971, retention index [RI]: 0.792), the newly derived sequence forms a clan (equivalent to a monophyletic group in a rooted tree; see Wilkinson, McInerney, Hirt, Foster, & Embley, 2007) with the archetypal sequence and a sequence derived from *H. medicinalis *(bootstrap support [BS] = 100%). Although the length of the branch leading to the two allies is relatively long, this result suggests orthology between the new sequence and true eglin C.

Much the same scenario is recovered for the tree resulting from the bdellin alignment (Figure 7b; length: 220, CI: 0.879, RI: 0.797) in which the new sequence forms a clan (BS = 100%) with the archetypal sequence, as well as several bdellin orthologues derived from *Haemadipsa interrupta* (Moore, 1935), *Macrobdella decora* Say, 1824,* H. medicinalis*, and *H. nipponia*. Again, the length of the branches within this clan is relatively long, but still portrays an image of orthology between the newly derived sequence and bdellin *sensu stricto*.

Due to the paucity of comparative data, only four sequences were included in the granulin alignment. Despite this, our newly derived sequence forms a clan with the archetypal sequence (BS = 100%) in the tree (Figure 7c; length: 173, CI: 1.00, RI: 1.00). This strongly suggests that our newly derived sequence is indeed an ortholog of granulin.

The tree resulting from the factor Xa inhibitor dataset (Figure 7d; length: 1,093, CI: 0.837, RI: 0.649) shows a tight grouping between the newly derived sequence and the archetypal sequence for guamerin, as well as a putative ortholog derived from *Aliolimnatis fenestrata* (Moore, 1939; BS = 100%). This suggests that the sequence derived from *W. pigra *is indeed a guamerin ortholog and, because of the inclusion of several other factor Xa inhibitors, we can rule out an affinity to any of the other known anticoagulants (e.g., antistasin, therostasin, ghilanten).

The hyaluronidase dataset included exclusively sequences labeled as mannilase. In the resulting tree (Figure 7e; length: 906, CI: 0.959, RI: 0.825), the newly acquired sequence forms a clan with the archetypal sequence (BS = 100%), with a relatively short branch length. This strongly suggests orthology between the two sequences.

A potentially different scenario is recovered for the destabilase I tree (Figure 7f; length: 521, CI: 0.769, RI: 0.618) wherein the newly acquired sequence nests with two putative orthologous sequences derived from *M. decora* and this clan does not include the archetypal sequence (BS < 50%). However, this clade is the adjacent group (equivalent to sister group relationship in a rooted tree; see Wilkinson et al., 2007) to a clan including the archetypal sequence. The clan constructed from these two subclans shows 100% bootstrap support. It therefore seems likely that all of the sequences included in this larger clan are orthologous with destabilase I. This is further supported by the strong sequence similarity seen in the alignments mentioned above.

Finally, the lipocalin tree (Figure 7g; length: 199, CI: 1.00, RI: 1.00) included only four putatively orthologous sequences, due to the dearth of comparative data for leeches. In fact, no “archetypal sequence” was included in the dataset, because this protein has seldom been sequenced for leeches. Our newly acquired sequence forms a tight clan (BS = 100%) with a hypothetical protein derived from *H. robusta*. Upon further inspection of this sequence, the protein product is labeled “lipocalin”, and this annotation is likely derived from sequence similarity with human‐ or mouse‐derived versions of this protein. The tree suggests orthology between the new sequence and the *H. robusta *version, but it is still unclear whether or not these are indeed lipocalin orthologs.

### 3.4 Differential gene expression

**Figure 8:**
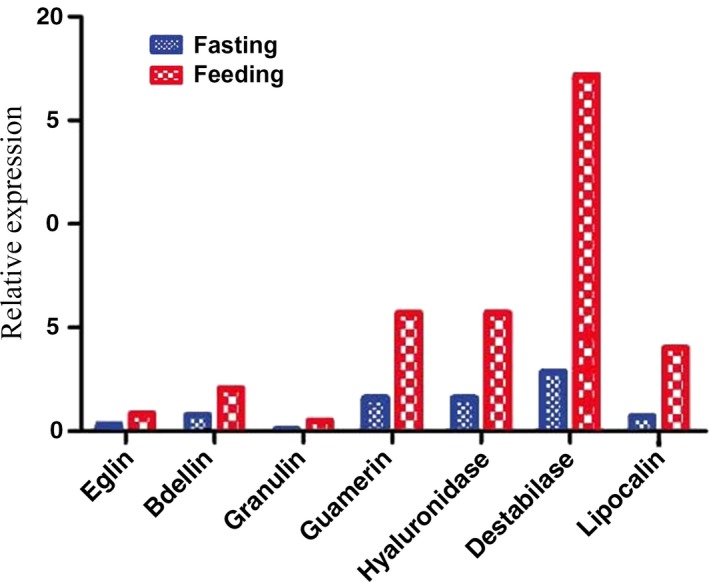
Relative expression of the seven putative anticoagulation factors as evinced by the quantitative real‐time PCR analysis. Red color indicates the expression of genes in leeches that were allowed to feed, and blue color indicates the expression of genes in fasting leeches

The DESeq in silico analysis identified strong evidence for the differential expression of proteins relating to several immunological pathways, but three pathways showed a distinctly higher number of differentially expressed genes: endocytosis, lysosome regulation, and peroxisome regulation (Supporting Information Table S2). Each of these pathways was recovered as statistically significantly (*p* < 0.05) upregulated when compared to other pathways. In total, 58 genes were found to be differentially expressed between the fasting and feeding modes, 31 of which were upregulated and 27 downregulated in the feeding mode of the leech. Seven of the 58 upregulated genes have been directly linked to anticoagulation and the expression levels of these were therefore further scrutinized using qRT‐PCR. For each of the putative anticoagulation proteins, leeches that were allowed to feed continuously show a marked increase in expression levels in the qRT‐PCR analysis (Table 2; Figure 8). The normalized log2 fold (see Love, Huber, & Anders, 2014) expression level change between the anticoagulation factors from feeding and fasting modes range between 0.3 and 3.1, suggesting an upper limit of 800% upregulation of the expression.

## 4 DISCUSSION

Through whole‐body transcriptomics and qRT‐PCR of known anticoagulation factors, our results indicate that (a) anticoagulation factors are expressed and secreted by the nonblood feeding leech *W. pigra* and (b) expression of several genes, most dramatically the anticoagulation factors, seems to be up‐ and/or downregulated depending on if the leech is in feeding or fasting mode. We found sequences with putative orthology to seven anticoagulation factors in our transcriptomic dataset—eglin C, bdellin, granulin, guamerin, hyaluronidase, destabilase I, and lipocalin. These factors antagonize the coagulation cascade in diverse ways, suggesting that *W. pigra *is capable of relatively effective anticoagulation. For example, eglin C is an inhibitor of elastase and cathepsin G (Baici & Seemüller, 1984; Seemüller, Fritz, & Eulitz, 1981), which are both involved in inflammatory processes and blood clotting disorders. Guamerin and bdellin act as both elastase inhibitors and as inhibitors of trypsin‐like serine protease inhibitors, thereby inhibiting the conversion of prothrombin to thrombin (Jung et al., 1995; Kim, Hong, Ha, Joe, & Kang, 1996; Salzet, 2001). Previous studies have shown putative orthology between guamerin and other strictly factor Xa inhibitors (e.g., Kvist et al., 2014), which is why this sequence was included in the factor Xa dataset used herein. With much the same result, destabilase I is capable of cleaving fibrin cross‐links (Salzet, 2001). By contrast, little is known about leech hyaluronidases, except that they are capable of cleaving hyaluronic acids (Kordowicz, Gussow, Hofmann, Pacuszka, & Gardas, 2006) and are on occasion mentioned as possible treatments for glaucoma (Min et al., 2010). In vitro studies of hyaluronic acid have demonstrated its potential of binding to collagen, the result of which is an increased coagulatory capability of the complex (Wu et al., 2008). Its specific utility in the salivary cocktail of leeches has yet to be realized, but it is likely that hyaluronidase performs similar tasks in leech saliva. Calin acts antagonistically against the coagulation cascade by blocking von Willebrand factor‐mediated binding of platelets to subendothelial collagen, thereby preventing the secretion of a granular matrix by collagen (Siddall et al., 2011). This is very similar to the function of leech antiplatelet proteins (LAPPs) and saratin (Kvist et al., 2011; Salzet, 2001), both of which have often been recorded from leech salivary extracts. Granulin, much like hirudin, directly inhibits thrombin and has previously been recovered from the hirudinid leech *H. nipponia *(Hong & Kang, 1999). The molecule is rather rich in cysteine residues (*n* = 12), suggesting a more complex folding structure than hirudin (*n* = 6). It is still unknown whether or not granulin mimics the irreversible effects of hirudin on the fibrin synthesis pathway by binding to both the catalytic site and the exosite of thrombin. During a normal thrombus formation, collagen, ADP, and TXA_2_ act in concert to activate signaling pathways and generating secondary messengers, all of which leads to the expression of integrins (Francischetti, Ribeiro, Champagne, & Andersen, 2000). Lipocalin, which was also recovered in our analyses of *W. pigra, *specifically binds to ADP, thereby halting the ADP‐induced pathways of platelet aggregation (Francischetti et al., 2000). Lipocalin has previously been recovered from a variety of blood feeding and nonblood feeding organisms (Fransischetti, 2010; Koh & Kini, 2008).

In combination with our knowledge of leech phylogenetics (e.g., Siddall et al., 2011; Tessler, de Carle et al., 2018), our results suggest that nonblood feeding leeches possess anticoagulation factors by virtue of their presence in a common ancestor. This hypothesis is gaining traction among hirudinologists and our study greatly increases our knowledge of the salivary components of nonblood feeding leeches; only very few studies have investigated the salivary peptide cocktails of nonblood feeding leeches (e.g., Kvist et al., 2011; Liu et al., 2018). The phylogenetic placement of *Whitmania *inside Hirudinidae and, further, rendering the genus *Hirudo *paraphyletic by virtue of nesting within (de Carle, D., Oceguera‐Figueroa, A., Tessler, M, Phillips, A. J., Siddall, M. E. & Kvist, S. Unpublished data), may be suggestive of a relatively recent transition from blood feeding to a macrophagous lifestyle. To this end, one could argue that *W. pigra* should maintain a higher expression level of anticoagulants than, for example, the distantly related *H. robusta* (see Kvist et al., 2011). This hypothesis is corroborated by the present study, due to the finding of seven putative anticoagulation factors as compared to only a single anticoagulant (LAPP) robustly inferred to be expressed by *H. robusta*.

Our resulting amino acid alignments and phylogenetic analyses suggest a close affinity between the seven anticoagulants found in *W. pigra *and the “archetypal” anticoagulants isolated from a range of other leech species. The phylogenetic analyses are vital in uncovering potential paralogues among the sequences, yet it is troublesome that no obvious outgroups exist for leech anticoagulation factors, such that no root can be applied. Therefore, it is difficult to decipher the direction of the evolution of the amino acid structures within leeches. However, it seems safe to say that, from our phylogenetic analyses, the anticoagulation factors expressed and secreted by *W. pigra *are orthologous with their archetypal counterparts.

Beyond the recovered anticoagulation factors, we also found significant matches against proteins that are utilized in important immunological pathways. Results from our in silico analyses of differential expression suggest that a disproportionate amount of genes relating to the following pathways show a distinct upregulation in all leeches, both feeding and fasting individuals: endocytosis, lysosome activity, and peroxisome activity. Lysosome plays an important role in the cytoplasm, where it is used to digest unwanted material and defective organelles. Whereas extracellular waste material is transported into the cell by endocytosis, unwanted intracellular components are transported to the lysosome via autophagy. In essence, lysosome acts as the cells immune system, removing unwanted or detrimental molecules, and is also valuable for cell homeostasis (Roczniak‐Ferguson et al., 2012). The foremost function of peroxisome is the disintegration of long‐chain fatty acids through beta‐oxidation. In animal cells, long‐chain fatty acids are changed into medium‐chain fatty acids and then transported to mitochondria where they are converted into carbon dioxide and water (Mannaerts, Debeer, Thomas, & De Schepper, 1979 and references therein).

To our knowledge, this is the first study to target differential expression of anticoagulant proteins in feeding and fasting leeches. Our results provide insight into the upregulation of anticoagulation factors during feeding and, in addition, provide some of the first data on the expression and secretion of anticoagulation factors by the nonblood feeding hirudinid *W. pigra*. Given our results, future investigations into the anticoagulation repertoires of leeches would benefit from including recently fed leeches, in order to recover as substantial a part of the expressed salivary transcriptome as possible.

## CONFLICT OF INTEREST

The authors declare that there are no competing interests.

## AUTHOR CONTRIBUTIONS

MSK and SQX designed the experiments. MSK and GDL performed the experiments. JYX, LBM, and SK analyzed the data. MSK wrote the manuscript draft. MSK and SK revised the manuscript.

## DATA AVAILABILITY

All clean data for each of the six *Whitmania pigra* specimens (fasting: *n* = 3; feeding: *n* = 3), and annotated data (CDS, SNP, etc.) are deposited in the NCBI Short Read Archive (Bioproject ID PRJNA523919).

## ETHICS APPROVAL

Not applicable.
