# Supplementary figures and images for: Transcriptomics and differential gene expression in Whitmania pigra (Annelida: Clitellata: Hirudinida: Hirudinidae): Contrasting feeding and fasting modes
Source: Ecol Evol. 2019 Mar 18;9(8):4706–19. doi: 10.1002/ece3.5074 (PMC6476756; doi:10.1002/ece3.5074)

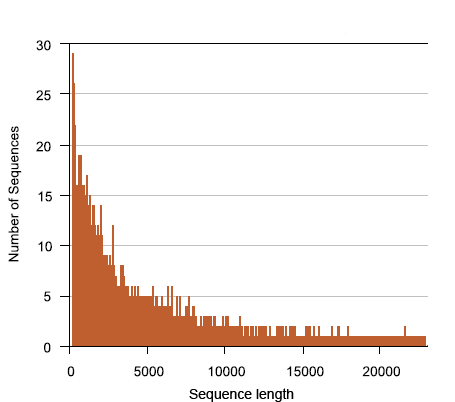

Supplement: Supplementary file 1 [file ECE3-9-4706-s001.tif]
