# Supplementary material for: Transcriptomics and differential gene expression in Whitmania pigra (Annelida: Clitellata: Hirudinida: Hirudinidae): Contrasting feeding and fasting modes
Source: Ecol Evol. 2019 Mar 18;9(8):4706–19. doi: 10.1002/ece3.5074 (PMC6476756; doi:10.1002/ece3.5074)
Supplement: Supplementary file 2 [file ECE3-9-4706-s002.docx]

**Supplementary Table 1.** Primers used for the qRT_PCR of the select, putative anticoagulation factors.

| **Gene** | **Forward primer (5`-3`)** | **Reverse primer (3`-5`)** |
| --- | --- | --- |
| **Lipocalin** | GCGATTCGTCTCCGTTTGTG | TCCATCAACGACGCTTGTGT |
| **Granulin** | TCCTTTTGCGAGAACGGTCA | AGGTCGTTGTGGACTCCTCT |
| **Hyaluronidase** | GCTTGCAATCTGCAACGAAGA | GTGCACGGATATTCACAACCA |
| **Bdellin** | GCACGAAGGAACTGAGACCA | GTGATGTTCCCCACCCAGAC |
| **Eglin** | TCGTTGGCAAAGCCGTAGAA | CGTGTCGTAAAACACACGCA |
| **Destabilase** | AGCCCTACTGGATCGACTGT | CGCCATTGTGGATTTTGGCA |
| **Guamerin** | GCTTGCAATCTGCAACGAAGA | GTGCACGGATATTCACAACCA |
