# Supplementary material for: Transcriptomics and differential gene expression in Whitmania pigra (Annelida: Clitellata: Hirudinida: Hirudinidae): Contrasting feeding and fasting modes
Source: Ecol Evol. 2019 Mar 18;9(8):4706–19. doi: 10.1002/ece3.5074 (PMC6476756; doi:10.1002/ece3.5074)
Supplement: Supplementary file 3 [file ECE3-9-4706-s003.docx]

**Supplementary Table S2.** Summary statistics for the DESeq analysis of differential gene expression. Each of the pathways mentioned possess at least a single gene that is up-regulated in all leech specimens. Note the proportionally high number of genes relating to the three pathways Lysosome, Peroxisome and Endocytosis.

| Pathways | # DEG in pathway | # genes in pathway | p-value |
| --- | --- | --- | --- |
| Leukocyte transendothelial migration | **1** | **46** | **0.95** |
| Thyroid hormone signaling | **1** | **28** | **0.84** |
| Spliceosome | **5** | **256** | **0.99** |
| Lysosome | **23** | **247** | **0.05** |
| Platelet activation | **1** | **39** | **0.93** |
| Inflammatory mediator regulation of TRP channels | **1** | **22** | **0.77** |
| Drug metabolism - cytochrome P450 | **2** | **40** | **0.75** |
| Peroxisome | **14** | **115** | **0.02** |
| Phagosome | **8** | **213** | **0.97** |
| Endocytosis | **25** | **276** | **0.05** |
| Bile secretion | **2** | **14** | **0.23** |
| Hedgehog signaling | **7** | **68** | **0.15** |
